# Next-Generation Sequencing Advances the Genetic Diagnosis of Cerebral Cavernous Malformation (CCM)

**DOI:** 10.3390/antiox11071294

**Published:** 2022-06-29

**Authors:** Valerio Benedetti, Rosalia Canzoneri, Andrea Perrelli, Carlo Arduino, Andrea Zonta, Alfredo Brusco, Saverio Francesco Retta

**Affiliations:** 1 Department of Clinical and Biological Sciences, School of Medicine and Surgery, University of Torino, Regione Gonzole 10, Orbassano, 10043 Turin, Italy; valerio.benedetti@unito.it (V.B.); rosalia.canzoneri@edu.unito.it (R.C.); andrea.perrelli@unito.it (A.P.); 2 CCM Italia Research Network, National Coordination Center at the Department of Clinical and Biological Sciences, University of Turin, Orbassano, 10043 Turin, Italy; 3 Department of Pharmacology and Physiology, University of Rochester Medical Center, Rochester, NY 14642, USA; 4 Medical Genetics Unit, Città della Salute e della Scienza University Hospital, 10126 Turin, Italy; carduino@cittadellasalute.to.it (C.A.); andrea.zonta@unito.it (A.Z.); 5 Department of Medical Sciences, University of Turin, 10126 Turin, Italy; alfredo.brusco@unito.it

**Keywords:** cerebrovascular disease, cerebral cavernous malformation (CCM), next-generation sequencing (NGS), clinical exome sequencing (CES), *KRIT1*/*CCM1*, aberrant splicing, genetic modifiers

## Abstract

Cerebral Cavernous Malformation (CCM) is a cerebrovascular disease of genetic origin that predisposes to seizures, focal neurological deficits and fatal intracerebral hemorrhage. It may occur sporadically or in familial forms, segregating as an autosomal dominant condition with incomplete penetrance and highly variable expressivity. Its pathogenesis has been associated with loss-of-function mutations in three genes, namely *KRIT1* (*CCM1*), *CCM2* and *PDCD10* (*CCM3*), which are implicated in defense mechanisms against oxidative stress and inflammation. Herein, we screened 21 Italian CCM cases using clinical exome sequencing and found six cases (~29%) with pathogenic variants in CCM genes, including a large 145–256 kb genomic deletion spanning the *KRIT1* gene and flanking regions, and the *KRIT1* c.1664C>T variant, which we demonstrated to activate a donor splice site in exon 16. The segregation of this cryptic splicing mutation was studied in a large Italian family (five affected and seven unaffected cases), and showed a largely heterogeneous clinical presentation, suggesting the implication of genetic modifiers. Moreover, by analyzing ad hoc gene panels, including a virtual panel of 23 cerebrovascular disease-related genes (Cerebro panel), we found two variants in *NOTCH3* and *PTEN* genes, which could contribute to the abnormal oxidative stress and inflammatory responses to date implicated in CCM disease pathogenesis.

## 1. Introduction

Cerebral Cavernous Malformation (CCM) is a major cerebrovascular disease with a known genetic origin (MIM# 116860, # 603284 and # 603285). It is characterized by the formation of abnormally dilated and fragile capillaries, which are devoid of normal vessel structural components, such as pericytes and astrocytic foot processes, and are referred to as CCM lesions [1,2,3]. These lesions (also known as cavernous angioma or cavernoma) mainly occur in the central nervous system (CNS), where they may remain clinically silent for a lifetime or unpredictably cause mild to severe clinical symptoms, including recurrent headaches, focal neurological deficits, seizures and fatal intracerebral hemorrhages (ICH) [1,2,3,4].

CCM disease has a prevalence of 0.5% worldwide and can occur in either sporadic (sCCM) or familial (fCCM) forms, the latter being inherited as an autosomal-dominant condition with incomplete penetrance and highly variable expressivity. Diagnosis is commonly made by magnetic resonance imaging (MRI), although detection is far more likely via gradient-echo (GRE) or susceptibility-weighted imaging (SWI), which can unmask small lesions [5]. CCM lesions can be single or multiple (even hundreds) and may range in size from a few millimeters to a few centimeters. Neuroradiological studies revealed that up to 70% of individuals harboring either single or multiple CCM lesions remain asymptomatic [1,2,3,5], suggesting that additional factors are necessary for disease exacerbation onset and progression. Currently, no therapies other than surgical removal of accessible lesions are available [6,7].

Sporadic cases of CCM are characterized by a lack of family history of the disease and usually the presence of a single lesion on MRI. In contrast, familial cases mostly exhibit multiple lesions of various size. Genetic studies conducted over the last 20 years have demonstrated that the hereditary form of CCM is caused by germline heterozygous loss-of-function mutations in one of three known genes: *KRIT1* (*CCM1*), *CCM2* and *PDCD10* (*CCM3*) [8,9,10,11,12,13]. On the other hand, recent whole-exome sequencing (WES) studies have shown that sporadic CCMs are mainly caused by somatic mutations in either phosphatidylinositol 3-kinase catalytic subunit alpha (*PIK3CA*) or mitogen-activated protein kinase kinase kinase 3 (*MAP3K3*) genes, whereas somatic mutations in three known CCM genes are comparatively very rare, thus defining a new subclass of CCM (CCM4, MIM#619538) [14,15,16].

A wide spectrum of pathogenic variants has been identified within each of the three known CCM genes, including nonsense mutations, deletions, insertions and intronic mutations leading to abnormal splicing and frameshift, which mostly lead to a premature stop codon [13]. In addition, there is also evidence for the existence of some alternative splicing variants that may generate protein isoforms with specific subcellular compartmentation and distinct expression patterns among various tissues and cells, suggesting that their abnormal expression may also contribute to CCM disease pathogenesis [17,18]. However, no mutation in either of the three known CCM genes were found in 5–15% of all CCM cases with a positive family history, suggesting either the potential existence of additional CCM genes or the presence of causative variants lost by traditional sequencing methods [12,19]. Moreover, whereas an incomplete penetrance and no clear genotype-phenotype correlations have been observed to date, the wide variability in phenotypes seen among carriers of the same CCM gene mutation, such as the development of CCM lesions of various numbers and sizes, and the occurrence and recurrence of ICH, suggests that the influence of additional pathogenicity determinants, including genetic and/or environmental modifiers [20,21,22,23,24]. In particular, growing evidence points to microenvironmental stress factors, such as oxidative stress and inflammation, as crucial determinants of CCM disease pathogenesis and severity, suggesting a potential impact of inter-individual heterogeneity in susceptibility to oxidative stress and inflammatory events due to genetic modifiers [22,24,25,26,27,28,29,30,31,32,33,34]. Consistently, we have previously shown that loss-of-function of CCM genes exerts pleiotropic effects on key redox-sensitive mechanisms involved in cellular homeostasis and defenses against oxidative stress and inflammation, leading to enhanced endothelial cell susceptibility to oxy-inflammatory insults that may contribute to the development of CCM lesions [22,24,34,35]. In particular, loss-of-function mutations of CCM genes result in a significant increase in the intracellular levels of reactive oxygen species (ROS) due to various mechanisms, including increased activation of mTOR (mechanistic target of rapamycin) signaling and consequent defective autophagy [27,28,29] and the upregulation of NADPH oxidase activity [30], as well as in the activation of major redox-sensitive transcription factors such as AP-1 [26], NF-κB [30] and Nrf2 [31,32]. Furthermore, genome-wide association studies (GWAS) in a large and homogeneous cohort of fCCM cases carrying the same disease-associated *KRIT1* mutation showed that polymorphic genes involved in oxidative stress and inflammatory responses, including distinct members of cytochrome P450 (CYP), matrix metalloproteinase (MMP) and Toll-like receptor (TLR) families, act as genetic modifiers of major disease severity phenotypes, such as the development of numerous and large CCM lesions and susceptibility to ICH [20,21,35].

Overall, accumulated evidence suggests that a combination of analyses, including sequencing and copy number analysis of germline DNA, and the assessment of the impact of disease-causative mutations at both the RNA and protein levels, is required to ensure that the best diagnostic sensitivity in detecting pathogenic variants in CCM genes and determining their pathogenic effects, as well as to identify genetic factors that modify clinical onset and severity of CCM disease. To this end, the rapid advances in high-throughput sequencing technologies, such as next-generation sequencing (NGS), offer the advantage of allowing the simultaneous detection of sequence variations in both disease-causing genes and genetic modifiers of disease penetrance and expressivity, thus providing reliable factual information of diagnostic and prognostic value to build up a better basis for an effective data-driven optimization of decision making in genetic counselling and clinical management.

Herein, we report the outcomes of our NGS screening on 21 Italian independent CCM cases, including the identification of distinct mutations in CCM genes and other genes potentially involved in CCM disease, and provide clinical and molecular genetic data on a large Italian family carrying the c.1664C>T splicing variant, characterized by broad heterogeneity in clinical presentation.

## 2. Materials and Methods

### 2.1. Patients and Clinical Assessment

Genetic analyses were performed on 21 Italian cases (12 females and 9 males) clinically affected by familial CCM, referred to the University Hospital “Città della Salute e della Scienza” of Torino. The diagnosis was performed following neuroradiological criteria for CCM [5,36,37]. Neuroradiological analyses and related clinical assessments were performed at the Department of Neuroscience of the University Hospital “Città della Salute e della Scienza” of Torino, Italy, by expert neuroradiologists and neurologists with twenty years of experience in the field [38], according to standard diagnostic neuroradiology and neurology procedures, including MRI of the brain and spine, and internationally accepted ethical guidelines for biomedical research. Magnetic resonance images were obtained with a 1.5 Tesla (T) MRI scanner using either spin-echo (SE) or gradient-echo (GE) pulse sequences. Written informed consent for participation and publication of data in biomedical journals, including case details and any accompanying images, was obtained from all subjects or their legal guardians, in accordance with the guidelines of the University of Torino Ethics Committee (ethics code: 0053916).

### 2.2. Molecular Genetic Analyses

NGS analyses were performed using a clinical exome sequencing (CES) panel by SOPHiA GENETICS (Saint-Sulpice, Switzerland). This approach and the relative optimized experimental procedure, including proband identification, library preparation, data analysis and variant interpretation, are described in detail in a previous report [39]. Briefly, genomic DNA (gDNA) was extracted from whole blood samples using the automated Maxwell^®^ 16 DNA Purification Kits set to the LEV (low elution volume) configuration; gDNA was purified using paramagnetic particles (PMPs), which provide a mobile solid phase that optimizes gDNA capture, washing and elution. Individual libraries were prepared using the DNA Library Prep Kit, AMPure XP^®^ (Beckman Coulter, Inc., Brea, CA, USA) beads and individual adapters provided by SOPHiA GENETICS as part of the Custom Solution kit. DNA was quantified by a Nanodrop spectrophotometer (Thermo Fisher Scientific, Waltham, MA, USA) and Qubit fluorometer (Thermo Fisher Scientific). CES was then performed using a NextSeq 500 instrument (Illumina, San Diego, CA, USA). Genetic raw data (∗.bcl files) generated by this instrument were converted to ∗.fastQ files using the bcl2fastq module provided by Illumina. Sequence alignments were performed with the PEPPER variant calling technology provided by SOPHiA DDM^®^ software platform (version 5.2) (SOPHiA GENETICS, Saint Sulpice, Switzerland). Single nucleotide variations (SNVs), small insertions/deletions (INDELs) and copy number variations (CNVs) were identified using the ILL1XG1G2_CNV/v5.3.5/GEN1GN1GN1FSQ2 pipeline, which was optimized by SOPHiA GENETICS for analyzing germline samples using the HPD-v1 kit. Identified variants were considered attenable with a sample coverage per run >25×, according to previous studies showing that a sequencing depth of 15–20× is an appropriate compromise of singleton detection power and sample size for studies of rare variants in complex disease [40]. Variant calling was performed with reference to the human genome version hg19/GRCh37 and using associated databases from ANNOVAR [39]. Sequence variations were annotated using SOPHiA PEPPER and MOKA technologies (SOPHiA GENETICS, Saint Sulpice, Switzerland), which include annotations following the recommendations of the Human Genome Variation Society, i.e., nucleotide position +1 corresponds to the A of the ATG translation initiation codon in coding cDNA reference sequences (GenBank, https://www.ncbi.nlm.nih.gov/gene, accessed on 8 October 2021). All the genetic variants that were present in less than 1% of the individuals in the control group and not reported in population databases (GnomAD version 2.1) were further considered to apply ACMG criteria [41]. All significant SNVs and CNVs identified by NGS were confirmed by Sanger sequencing or real-time PCR on a second genomic DNA extraction. The co-segregation of disease phenotypes and detected variants was confirmed in all affected family members by PCR amplification and Sanger sequencing using an ABI PRISM^®^ 3130xl Genetic Analyzer (Applied Biosystems, Thermo Fisher, Waltham, MA, USA). Sanger sequencing to confirm the *KRIT1* c.1664C>T mutation was performed with a set of primers specific for exon 16 (forward: 5′-gcttttcccatattaagttgttcatt; reverse: 5′-ttggttaaacagaatcttaagcatagc) using the KAPA2G Fast HS PCR kit (Kapa Biosystems, Bath, UK) and ABI PRISM^®^ 3130xl Genetic Analyzer (Applied Biosystems, Foster City, CA, USA), according to the manufacturer’s recommendations.

### 2.3. Quantitative Real-Time PCR

Gene-specific real-time expression-quantification assays were designed using the web-based Universal ProbeLibrary System Assay Design (Roche Life Science), including a set of primers and probes specific for *KRIT1* (exons 4, 13, 19) (RefSeq: NM_194456.1), *AKAP9* (introns 16 and 38) (RefSeq: NG_011623.1), *CYP51A1* (exon 1) (RefSeq: NM_000786.3) and *ANKIB1* (exon 6) (RefSeq: NM_019004) (Supplementary Table S1).

The real-time PCR assays were performed on a 7500 Fast Real-Time PCR System (Applied Biosystems, Thermo Fisher, Waltham, MA, USA) in a 20 μL total volume of a reaction mixture of containing 2X TaqMan Universal PCR Master mix (Life Technologies, Carlsbad, CA, USA), 1X RNase P assay (20X, VIC dye), 0.2 μM sequence-specific forward and reverse primers and 0.1 μM TaqMan probe. The PCR cycling conditions included an initial denaturation of 95 °C for 3 min followed by 40 cycles of 95 °C for 15 s and 60 °C for 45 s. Each sample was analyzed in triplicate. The copy number was calculated using the comparative delta Ct method.

### 2.4. RNA Isolation, cDNA Synthesis and KRIT1 Transcript Analysis

Total RNA was isolated from peripheral blood samples using the PAXgene Blood RNA Isolation System (PAXgene), consisting of the PAXgene Blood RNA Tube and the PAXgene Blood RNA purification kit, according to the manufacturer’s protocol (PreAnalytiX GmbH, Hombrechtikon, CH, QIAGEN Group). Purified RNA samples were then quantified with NanoDrop and complementary DNA (cDNA) was synthesized from 2 μg of RNA template using the Transcriptor first strand cDNA synthesis kit from Roche (Roche Molecular Systems, Inc., Pleasanton, CA, USA). To assess the effect of the *KRIT1* c.1664C>T mutation at the mRNA level, cDNA was amplified by PCR using the following forward (F) and reverse (R) primers: 15F (5’-atctggatcctcaaagggaaac, corresponding to the nucleotide segment 1487–1509) and 17R (5’-ggtgccttacttttcagtttggt, corresponding to the nucleotide segment 1762–1785). The PCR product was separated by agarose gel electrophoresis, and individual bands were purified by the GenElute Gel Extraction Kit (Sigma-Aldrich, St. Louis, MO, USA) and sequenced by the Sanger method as described above. The cDNA bases were numbered according to the *KRIT1* reference sequence in GenBank NM_194456.1.

## 3. Results

### 3.1. NGS-Based Genetic Screening for CCM Disease

We collected 21 independent patients carrying either a single or multiple CCM lesions, as assessed through neuroradiological diagnosis by MRI. In most cases, CCM lesions were located in the brain; however, spinal and retinal lesions were also detected.

To perform our genetic survey, we used a clinical exome sequencing (CES) strategy by the Sophia Genetics DDM (Saint-Sulpice, Switzerland), which included 4490 disease-associated genes. This strategy has previously been optimized and described in detail [39].

The NGS data analysis was performed in two steps: initially we considered the three known *CCM* genes, namely *KRIT1* (*CCM1*), *CCM2* and *PDCD10* (*CCM3*), and subsequently extended the analysis to NGS panels, including the “Cerebro” panel composed of 23 genes associated with cerebrovascular diseases (Table 1). The outcomes of these analyses are summarized in Table 2.

**Figure 1:**
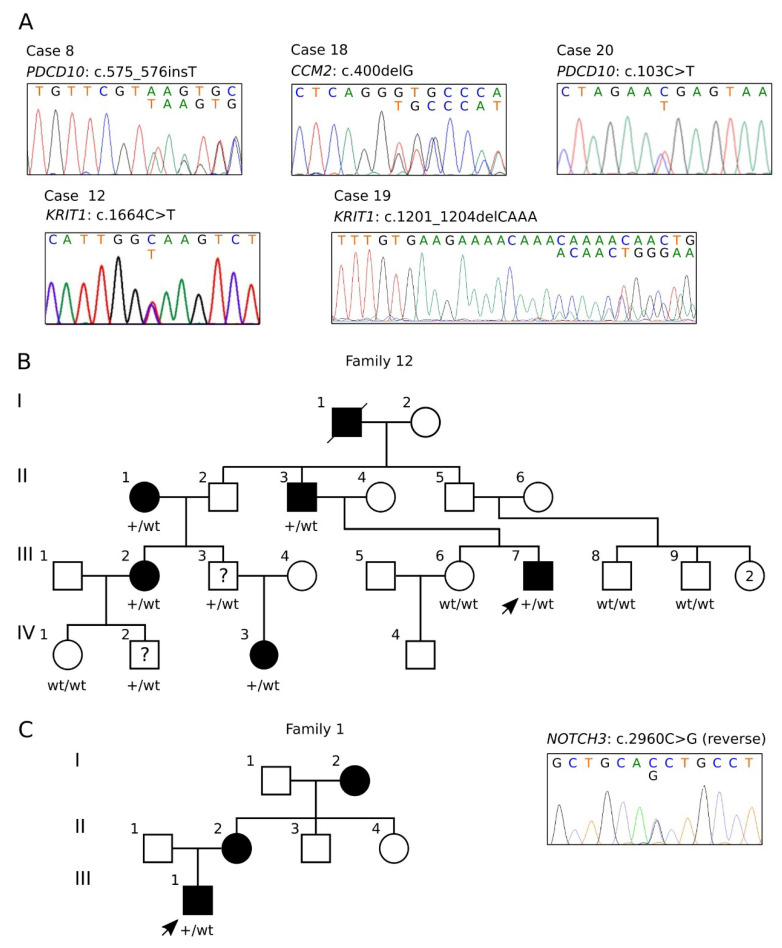
Sanger validation and pedigrees. (**A**) Electropherograms of the identified CCM gene variants reported in Table 2. (**B**) Four-generation pedigree of family 12. Filled symbols indicate affected subjects; a question mark indicates the subjects carrying the pathogenic variant but without signs of disease. The genotype is indicated below each tested subject (wt/wt, homozygous wild type; wt/+, heterozygote for the *KRIT1* c.1664C>T variant). (**C**) Pedigree of family 1 (left) and electropherogram of the identified *NOTCH3* c.2960C>G variant (right).

The identified SNV variants were confirmed by Sanger sequencing (Figure 1A) and data were submitted to ClinVar. In particular, we identified five likely pathogenic/pathogenic variants in CCM genes (Table 2, cases #8, 12, 18, 19, 2, and Figure 1A), one of which had never been reported before (*CCM2* c.400delG) (Table 2, case #18, and Figure 1A). Furthermore, we could demonstrate that the c.1664C>T variant identified in case #12 (Table 2 and Figure 1A) segregated in several family members within a four-generation pedigree (Figure 1B) and exhibited incomplete penetrance and highly variable expressivity (see below).

In addition, using the Cerebro panel, we found the interesting *NOTCH3* c.2960G>C p.(Thr987Ser) missense variant (Table 2, case #1), which was confirmed by Sanger sequencing and shown to segregate in two affected family members (Figure 1C), as discussed further below.

Finally, in case #4, which was affected by PTEN hamartoma tumor syndrome (PHTS) combined with multiple brain CCMs, we detected the c.959dup p.(Leu320PhefsTer5) frameshift variant in exon 8 of *PTEN* (NM_000314.8; LRG_311) with a known pathogenic clinical significance for PHTS (ClinVar ID: RCV000801646.1). Interestingly, in addition to this *PTEN* variant, which resulted hitherto unlikely to be directly linked to CCM phenotype, we did not find any additional variant possibly responsible for the multiple CCM lesions affecting case #4. However, given the well-established role of PTEN as a negative regulator of the PI3K/Akt signaling pathway, the very recent discovery that activating mutations in genes involved in such pathway are implicated in CCM disease pathogenesis [14,15,16,44] leaves open the intriguing possibility that *PTEN* mutations may indeed contribute to CCM disease, thus raising the necessity for further investigation.

### 3.2. Characterization of the KRTI1 Genomic Deletion Identified in Case #7

**Figure 2:**
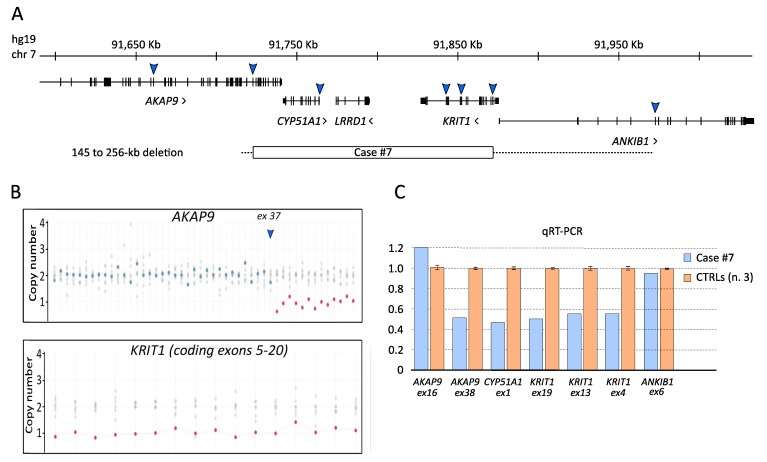
Analysis of the *KRIT1* genomic deletion identified by NGS in case #7. (**A**) Screenshot from UCSC genome browser centered on *KRIT1* (hg19). Arrows approximately indicate the genomic regions analyzed by real-time PCR assays in order to verify the deletion identified by NGS, including its chromosomal mapping and extension. The minimal extension of the identified deletion is indicated by a rectangle; the hyphened line indicates a region of uncertainty. (**B**) Exon dosage for *AKAP9* and *KRIT1* genes obtained by WES data. (**C**) Histogram showing the outcomes of quantitative real-time PCR (qRT-PCR) assays.

The NGS genetic screening for CCM disease allowed to demonstrate that case #7 was affected by a large hemizygous deletion on chromosome 7, involving the entire *KRIT1* gene and upstream flanking sequences, including the entire *CYP51A1* gene and the last 13 coding exons (from 38 to 50) of the *AKAP9* gene (Figure 2A,B), which was previously undetected by Sanger sequencing. This deletion was further verified by quantitative real-time PCR (qRT-PCR) on genomic DNA using multiple assays specific for *KRIT1* and adjacent genes, including the upstream *CYP51A1* and *AKAP9* genes and the downstream *ANKIB1* gene (Figure 2C). Specifically, the centromeric and telomeric deletion breakpoints were located between exon 37 (non-deleted) and exon 38 (deleted) of *AKAP9*, and between the first coding exon of *KRIT1* (exon 4, deleted) and exon 6 (non-deleted) of *ANKIB1*, respectively. Although we could not map the breakpoints at the nucleotide level, the size of the deletion was estimated in 145–256 kb based on the mapping of the maintained/deleted exon coordinates (NC_000007.13:g.(91715729_91718698)_(91864237_91972337)del).

### 3.3. The KRIT1 c.1664C>T Variant Leads to Aberrant Exon Skipping and Segregates with CCM Disease

**Figure 3:**
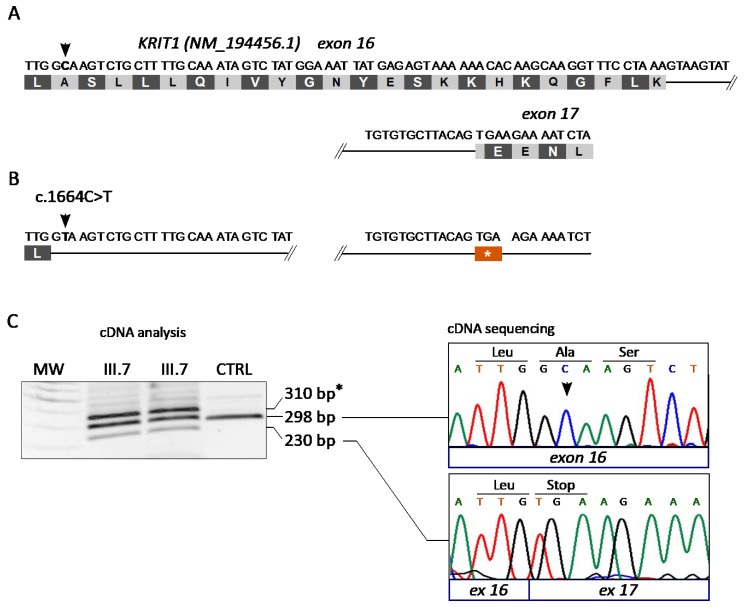
Molecular analysis of the *KRIT1* c.1664C>T variant. (**A**,**B**) Schematic representation of the *KRIT1* exons 16–17 region in wild-type (**A**) and c.1664C>T mutated (**B**) transcripts. (**C**) Molecular analysis of the *KRIT1* c.1664C>T change at the mRNA level. Total RNA isolated from peripheral blood samples of two affected members of family 12 (III.7 and II.1) and a control was reverse transcribed into cDNA and analyzed by PCR amplification with a pair of forward and reverse primers localized in exons 15 and 17 of *KRIT1*, respectively, followed by agarose gel electrophoresis (left panel) and Sanger sequencing (right panel) of purified PCR products. The resulting sequencing electropherograms (right panel) showed that the 298 bp and 230 bp bands corresponded, respectively, to the wild-type *KRIT1* transcript and to an anomalous splicing product devoid of the last 68 nucleotides of exon 16, which leads to the formation of a premature stop codon due to an in-frame TGA sequence in the three first nucleotides of exon 17. Asterisk (*) indicates a likely heteroduplex band.

In case #12, we found the *KRIT1* c.1664C>T variant. This variant was identified in a previous study as an apparent Ala555Val missense mutation predicted to affect splicing [43]. Indeed, according to Human Splicing Finder (version 3; http://www.umd.be/HSF/, accessed on 8 October 2021), the *KRIT1* c.1664C>T introduces a novel splice donor site within exon 16 (Figure 3A,B).

To address this hypothesis, we isolated RNA samples from the white blood cells of two affected members of family 12 (III.7 and II.1) and a control (CTRL) and performed a specific molecular analysis at the mRNA level. To this end, cDNA was generated by reverse transcription and analyzed by PCR and agarose gel electrophoresis using a pair of forward and reverse primers localized in exons 15 and 17 of *KRIT1*, respectively. The outcomes demonstrated the presence of three bands in samples deriving from both affected members of family 12 (III.7 and II.1), including the expected band of 298 bp that was present in the control (CTRL), and two additional bands of higher (~310 bp) and lower (230 bp) molecular weight (Figure 3C, left panel). The 298 bp and 230 bp bands were then purified from the gel and sequenced by the Sanger method, showing that they corresponded, respectively, to the wild-type *KRIT1* transcript and to an anomalous splicing product devoid of the last 68 nucleotides of exon 16 (Figure 3C, right panel). Such an aberrant transcript would lead to the formation of a premature stop codon at amino acid 555 due to an in-frame TGA stop codon in the three first nucleotides of exon 17 (Figure 3B,C, right panel). Overall, these findings confirmed that the *KRIT1* c.1664C>T mutation generates a splice-donor site in exon 16, leading to the aberrant skipping of a 3’ exon fragment of 68 bp, and the formation of a premature stop codon in the mRNA transcript. The variant was therefore designated as c.1664C>T; r.1663_1690del; p.Ala555Ter.

Genetic testing was then extended to all available family members. Overall, out of a total of 12 family members analyzed, 7 resulted positive (II.1, II.3, III.2, III.3, III.7, IV.3, IV.2) and 5 negative (I.2, III.6, III.8, III.9, IV.1) for the identified *KRIT1* c.1664C>T mutation (Figure 1B). Notably, while all cases affected by clinical and neuroradiological signs of CCM disease were confirmed to be heterozygous carriers for the *KRIT1* c.1664C>T variant, an adult male of 42 y (III.3) and a male child of 20 y (IV.2) were found to be carriers but they currently had no clinical symptoms and showed a normal MRI, thus confirming the incomplete penetrance of pathogenic germline mutations in *KRIT1*.

### 3.4. Clinical Features of Cases Carrying the KRIT1 c.1664C>T Variant (Family 12)

The pedigree of family 12 is shown in Figure 1B. This was a large Italian family from Piemonte region characterized by the distinct members of four consecutive generations affected by CCM lesions of various number and size in either the brain or spinal cord, or both. Co-segregation of the disease phenotype with the *KRIT1* c.1664C>T variant was confirmed by Sanger sequencing in all affected family members. Conversely, in addition to the absence of clinical and neuroradiological signs in the aforementioned III.3 and IV.2 mutation carriers, it is noteworthy that symptomatic mutation carriers displayed varying degrees of clinical severity.

**Figure 4:**
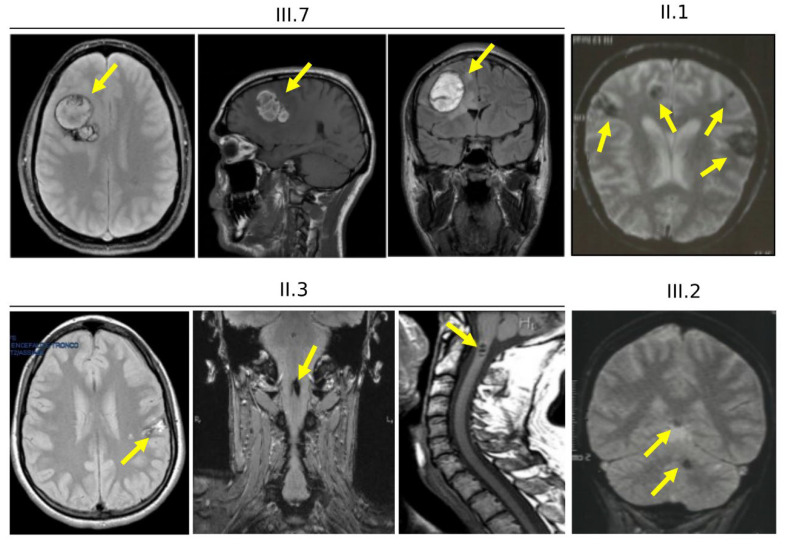
Brain and spinal cord magnetic resonance imaging of family 12. Representative brain and cervical spine MRI images of the proband (**III.7**) and three affected family members (**II.1**, **II.3** and **III.2**) obtained with a 1.5T MRI scanner. Arrows indicate CCM lesions (also see Figure 1B and Supplementary Figure S1).

The index patient (III.7) was an adult male suffering from tonic-clonic seizures, and treated with valproate and carbamazepine, two well-established antiepileptic drugs. Brain MRI revealed multiple and large CCM lesions in the CNS (Figure 4, III.7), mainly located in the left periventricular region and left frontal lobe and characterized by mild bleeding signs. No lesions were identified in the spinal cord.

The proband’s father (Figure 4, II.3) showed neurological symptoms associated with a spinal radiculopathy, loss of sensitivity to sensory stimuli and disability affecting the lower limbs, and epileptic seizures during the childhood. Both cerebral and spinal MRI were positive for CCM lesions (Figure 4, II.3). Brain MRI showed multiple small CCMs (up to 8 mm), located in the white matter of the right cerebellar hemisphere, and in the right frontal and parietal lobes. Gradient-echo (GRE) sequences demonstrated recent micro-bleeding in the parietal lesions (Figure 4, II.3). Spinal MRI showed multiple lesions located in the cervical, thoracic and lumbar spinal cord (Figure 4, II.3 and Supplementary Figure S1A–G), which caused various disorders, including motor dysfunction.

Furthermore, symptomatic CCM lesions of various size and number were also detected in the brain of both the proband’s paternal aunt (Figure 4, II.1 and Supplementary Figure S1H–L) and her daughter (Figure 4, III.2) and granddaughter (IV.3, not shown), but not in her son (III.3, not shown). Clinical symptoms ranged from headaches (III.2) to neurological deficits (IV.3) and recurrent bleeding (II.1). In addition, although no medical data were available, a detailed family history revealed that the proband’s paternal grandfather (I.1) deceased from a cerebrovascular disease, allowing to trace the path of CCM disease hereditary transmission in this large family.

### 3.5. Characterization of the NOTCH3 c.2960G>C Variant Identified in Case #1

The screening of the 23 genes of Cerebro panel allowed us to identify the *NOTCH3* c.2960G>C p.(Thr987Ser) variant in case #1. This variant (rs752995216) has a global frequency of 14/215,582 alleles (GnomAD 2.1.1) and a maximum frequency in South Asian of 4/27,588 (1/3450 carriers). The threonine 987 is located in EGF-like 25 extracellular domain between two cysteine residues which are predicted to form disulfide bonds. Bioinformatic software are contradictory in defining pathogenicity. The threonine in position 987 is a modestly conserved residue in vertebrates. The conservative substitution between two polar amino acids is considered of little relevance by the BLOSUM62 (score 2) and Grantham (score 58) matrices, whereas bioinformatics predictions favor either a potential benign effect (low impact substitution for Provean, SIFT, MutAssessor, LRT, DANN, A-GVGD and PolyPhen2) or a deleterious effect (the Thr 987 is intolerant to variation for Fathmm, MutTaster and MetaDome). Moreover, according to four different computer models of splicing simulation, the substitution c.2960C>G determines the activation of a cryptic splice acceptor site inside exon 18, with the consequent loss of 168 nucleotides. If this splicing event occurs, it would cause an in-frame deletion of 56 amino acids in the NOTCH3 protein, including some cysteine residues that play a key role in its function.

### 3.6. Clinical Features and Family History of Case #1

At 16 y, the patient complained of left arm and hand paresthesia associated with vomiting and dumbness, mainly triggered by stressful events the night before the symptoms. Brain MRI performed at 17 y showed a cerebral cavernoma (15 mm) in the right insula, which was surgically removed.

His mother (see pedigree in Figure 1C) was clinically healthy, but brain MRI at 54 y showed the presence of multiple CCMs in both cerebral hemispheres. Maternal grandmother had also multiple CCMs in the absence of specific clinical signs; however, its DNA was not available.

## 4. Discussion

CCM disease is a major cerebrovascular disorder of genetic origin that has been estimated to affect approximately 25 million people worldwide. It occurs in either sporadic (sCCM) or familial (fCCM) forms, and may cause various clinical symptoms, including severe seizures, neurological deficits and intracerebral hemorrhage (ICH) [4]. Sporadic cases often have a single CCM lesion, whereas the familial form is commonly characterized by multiple lesions. Although the exact prevalence of each form still remains unclear, estimated data from large clinical cohorts suggest that approximately 80% of CCM cases are sporadic and 20% are familial [13]. Up to 85–95% of familial cases have been shown to harbor a germline heterozygous loss of function (LoF) mutation in one of three identified causative genes, namely *KRIT1* (*CCM1*), *CCM2* and *CCM*3 [13]. In particular, *KRIT1* mutations account for more than 50% of the fCCM cases, whereas mutations in *CCM2* and *CCM3* genes account for approximately 20% and 10% of the fCCM cases, respectively. The remaining 5–15% of cases were not attributable to mutations in the three known CCM genes, suggesting the existence of yet unidentified causative genes [12,13]. To this regard, it is noteworthy that, in recent years, high-throughput sequencing technologies have helped to identify novel genetic variants of pathogenic significance underlying various diseases, which could be consistent with the experimental outcomes of our study, including the identification of *NOTCH3* and *PTEN* germline variants in fCCM cases devoid of mutations in known CCM genes. On the other hand, the genetic basis of sCCM cases can only rarely be ascertained, as the identification of any somatic mutations underlying such cases can only be carried out through the analysis of surgical specimens, if available. However, very recent studies, specifically those performed by distinct research groups on surgical samples of human CCM lesions, have led to the breakthrough discovery that somatic mutations in the three known CCM genes are rarely present in sporadic CCMs, which are instead most frequently associated with somatic mutations in genes implicated in the PI3K/Akt and MAPK pathways, including *PIK3CA* and *MAP3K3* [14,15,16]. Furthermore, concomitant studies in conditional knockout mouse models have shown that the growth of CCM lesions requires both the loss of function of a CCM gene and increased activation of the PI3K/Akt/mTOR pathway in the same endothelial cells [44]. Considered together, these findings support the notion that CCM disease can be caused by mutations in other genes besides the three CCM genes identified to date, as well as that a local upregulation of the PI3K/Akt and MAPK pathways induced by either somatic mosaic mutations in regulatory genes or microenvironmental events is likely to exert a key pathogenetic role.

Taking advantage of an Italian research network with a coordination center at the University of Torino, we previously performed the molecular genetic analyses of the three known CCM genes in a large Italian cohort of CCM patients and at-risk relatives using the traditional Sanger exon sequencing approach, showing that 67% the familial cases had a mutation in *KRIT1*, 5.5% in *CCM2* and 5.5% in *CCM3*, whereas no mutations in CCM genes were detected in the remaining 22% of cases [45]. More recently, we developed an optimized NGS-based genetic testing procedure for the high-throughput analysis of hereditary cerebrovascular disorders [39]. Indeed, NGS technology has now become technically feasible and cost-effective due to great advances in high-throughput sequencing methods and bioinformatics analysis, which have offered new opportunities for Mendelian disorder research, including the identification of causative or modifying genes and the discovery of rare structure and functional genetic variants, thus facilitating clinical diagnosis and personalized disease-risk profiling [46,47,48]. In particular, recent coverage and accuracy improvements have accelerated the development of clinical exome sequencing (CES) platforms targeting disease-related genes and enabling variant identification in patients with suspected genetic diseases [39]. CES analysis has in fact been introduced by several companies as a diagnostic-oriented tool able to cover the coding regions (±5 bp of intronic regions) of several thousands (5000–7000) of disease-associated genes. This approach is considered more effective than the whole genome and whole exome sequencing (WGS/WES) to provide a quick and cost-effective identification of pathogenic variants in human diseases, including single nucleotide variations (SNVs), small insertions/deletions (INDELs) and copy number variations (CNVs). Furthermore, they allow the Medical Genetics Units to have a unique method to analyze a wide spectrum of genetic diseases. On the other hand, genetic heterogeneity, phenotypic variability, and disease rarity are factors that make the traditional diagnostic approach to genetic disorders very challenging and obsolete [39], whereas the NGS technology has the further advantage of being easily adaptable to any changes in the diagnostic scenario [49]. This is particularly true and relevant for CCM disease, given the emerging evidence that variants in other genes besides *CCM1/KRIT1*, *CCM2* and *CCM3* are implicated, causing clinically indistinguishable phenotypes, and the concomitant necessity to search for both SNVs and CNVs in several exons.

Here, we report the outcomes of NGS analyses of a local case series of distinct Italian patients and families affected by CCM disease, which allowed the rapid identification of causative mutations in CCM genes, as well as of genetic variations in other genes that might play a role in CCM disease pathogenesis and severity. Specifically, we identified six different pathogenic variants of CCM genes in 6/21 (≈29%) cases, including two novel ones (Table 2). This detection rate depends on the choice of including both familial and sporadic cases, and is consistent with the fact that 70–80% of CCM cases are sporadic. Moreover, we found two interesting variants in *NOTCH3* and *PTEN* genes, which may contribute to the emerging pleiotropic mechanisms underlying CCM disease pathogenesis, thereby expanding the spectrum of potential causative genes and suggesting relevant implications for future research and diagnostic procedures. In addition, we reported the outcomes of subsequent in-depth clinical and molecular genetic studies of a large Italian family (family 12), whose numerous affected members were demonstrated to carry the *KRIT1* c.1664C>T germline mutation associated with a broad heterogeneity in clinical and neuroradiological presentation. In particular, the MRI analyses of distinct mutation carriers of this family revealed the presence of highly heterogeneous CCM lesions, in terms of localization to either the brain or spinal cord, number, size and susceptibility to ICH, which corresponded to a wide spectrum of clinical manifestations, ranging from an asymptomatic state or mild neurological symptoms to peripheral radiculopathy, severe seizures and cerebral bleeding, suggesting an incomplete penetrance and highly variable expressivity of the disease-causing mutation. Genetic analysis at the mRNA level demonstrated that the c.1664C>T change introduces a novel splice donor site within the exon 16 of *KRIT1*, leading to the aberrant skipping of its last 68 nucleotides and the formation of a premature stop codon. This disease-causative mutation was therefore designated as c.1664C>T; r.1663_1690del; p.Ala555Ter, and is predicted to result in a complete LoF. Nevertheless, the incomplete penetrance and variable expressivity observed in this large family clearly suggest that the identified *KRIT1* LoF mutation is not sufficient to cause CCM disease but requires the contribution of additional determinants. Accordingly, growing evidence points to a major pathogenic role of environmental insults, including oxidative-stress and inflammatory events [22,25,26,27,28,29,50,51], as well as to a significant contribution of inter-individual heterogeneity in susceptibility to such events [20,21,35]. Distinct genetic modifiers of endothelial cell responses to oxidative stress and inflammation, including polymorphic variants of distinct members of the CYP, MMP and TLR4 gene families, have been indeed reported to impact the severity of CCM disease [20,21,35,51]. Furthermore, whereas the evidence for a major role of *KRIT1* in redox signaling and cell responses to oxidative stress and inflammation continues to grow [24,52], recent findings demonstrate that the consequences of *KRIT1* LoF mutations may extend beyond CCM disease pathogenesis, also being implicated in atherosclerosis [53] and intestinal barrier dysfunction [54].

In addition to expanding the spectrum of CCM gene functions, these findings also provide fundamental insights into the identification of biomarkers of prognostic and predictive value for the effective risk stratification and clinical management of CCM patients [20,21,35,55,56], as well as into the development of preventive and therapeutic approaches [22,29,30,57,58,59,60,61,62,63]. Furthermore, they also raise the intriguing possibility that CCM lesions may develop independently of CCM gene mutations, mainly as a consequence of a combination between inter-individual genetic variability in sensitivity to oxy-inflammatory conditions and local increases in such conditions. Consistently, we obtained preliminary results that suggest a major role for pro-inflammatory microenvironmental conditions generated by cerebral developmental venous anomalies (DVAs) in the secondary formation of sporadic CCM lesions, which are indeed known to be frequently and peculiarly associated with DVAs. In this context, it is noteworthy that the possibility that sporadic CCM lesions may develop independently of CCM gene mutations is also supported by the recent evidence that somatic mutations in CCM genes are rarely detected in the surgical specimens of human sporadic CCM lesions [14,15,16].

In this light, SNVs and CNVs in other genes not yet directly associated with CCM disease, including those in *CYP51A1*, *AKAP9*, *NOTCH3* and *PTEN* genes identified by our NGS analysis might be unpredictably implicated in CCM disease pathogenesis and severity. In particular, whereas *AKAP9* is known to enhance endothelial barrier function [64], both *CYP51A1* and *AKAP9*, the two *KRIT1*-flanking genes comprised in the large hemizygous deletion identified in case #7, have been proposed as putative prognostic biomarkers associated with oxidative stress-related diseases [21,65], suggesting a potential role as genetic modifiers of CCM disease expression and severity, which deserves further investigation.

Moreover, the *NOTCH3* c.2960G>C variant, identified in case #1 and shown to segregate with CCM disease in two family members (Figure 1C), is present with very low frequency in human genetic variation databases, indicating that it is not a common polymorphism. This variant was previously identified in a family affected by Cerebral Autosomal Dominant Arteriopathy with Subcortical Infarcts and Leukoencephalopathy (CADASIL) [42], a rare hereditary cerebral small vessel disease associated with distinct low or incomplete penetrant *NOTCH3* mutations and characterized by abnormalities in arterioles and capillaries, including abnormal pericyte–endothelial interactions, which compromise BBB integrity [66,67,68]. Remarkably, whereas there is evidence that reduced endothelial cell–pericyte association and basement membrane alteration are integral parts of both CADASIL [68,69] and CCM [70,71] pathogenesis, the co-occurrence of CADASIL and CCM lesions has been also reported, suggesting a common pathogenetic mechanism driven by *NOTCH3* mutations leading to microangiopathy [72]. Consistent with this possibility, there is also evidence that CCM proteins control DLL4-NOTCH3 signaling between the endothelium and pericytes, which is important to maintain a quiescent vascular phenotype and prevent angiogenesis [73], suggesting that *NOTCH3* mutations causing deregulated DLL4-NOTCH3 signaling may indeed contribute to the pathogenesis of both CADASIL and CCM disease. To this regard, it is noteworthy that *NOTCH3* variants are more frequently present in the general population than expected from CADASIL prevalence and are considered genetic risk factors for common, multifactorial cerebral small vessel disease (cSVD) and related clinical manifestations, including cerebral microbleeds and apparently ‘sporadic’ stroke [74,75]. In this light, the recent discovery that *NOTCH3* is involved in the regulation of ROS and lipid peroxidation levels [76] raises the intriguing possibility that the pathogenic effects of *NOTCH3* mutations are somehow related to alterations in cellular redox homeostasis and defenses against oxy-inflammatory insults so far implicated in the pathogenic effects of loss-of-function mutations in CCM genes [22,34,35]. Remarkably, this possibility is also supported by the finding that aberrant NOTCH3 function promotes ROS production, oxidative stress and the activation of the RhoA/Rho kinase (ROCK) pathway, leading to major phenotypic features of CADASIL, including altered actin cytoskeleton organization and impaired vascular function [77]. In fact, the abnormal activation of the RhoA/ROCK pathway and consequent actin cytoskeleton alterations and vascular dysfunctions are also major hallmarks of CCM disease [78,79,80]. Furthermore, given the evidence that cells expressing mutant NOTCH3 develop increased sensitivity to stressful conditions, including oxidative stress [81], it is conceivable that *NOTCH3* mutations may contribute to the enhanced cell susceptibility to additive effects caused by oxy-inflammatory insults underlying the focal development of CCM lesions [20,22,34,35]. Accordingly, whereas the downregulation NOTCH signaling has been clearly linked to the loss of function of CCM genes [82,83,84], there is also evidence that such a downregulation is redox dependent and can be rescued by antioxidant treatment [53]. Overall, while further studies are required to address the intriguing possibility that incomplete penetrant *NOTCH3* mutations contribute to the pathogenesis not only of CADASIL but also of CCM disease, our finding that the *NOTCH3* c.2960G>C variant segregates with CCM disease paves the way forward to exploring its impact on molecular mechanisms involving CCM genes.

Finally, given the recent discovery that activating mutations in genes involved in the PI3K/Akt signaling pathway, including *PIK3CA* and *Akt*, are implicated in CCM disease pathogenesis [14,15,16,44], it would be intriguing to address whether the *PTEN* c.959dup p.(Leu320PhefsTer5) frameshift variant identified in case #4 may be also implicated. In fact, PTEN is a well-established negative regulator of PI3K-dependent Akt signaling, and its loss-of-function mutation or oxidative inactivation have been shown to cause the hyperactivation of Akt and consequent modulation of its downstream targets, including activation of mTOR and inhibition of FoxO1, leading to increased angiogenic responses and enhanced cell sensitivity to oxidative stress [85,86,87,88,89,90]. Remarkably, both mTOR activation and FoxO1 inhibition were originally demonstrated to be induced by CCM gene loss of function and associated with increased ROS levels and reduced antioxidant defenses via the downregulation of autophagy [27] and superoxide dismutase 2 (SOD2) [25], respectively. Moreover, as hyperactive PI3K/Akt signaling is known to contribute to increased ROS production and the subsequent induction of downstream ROS-dependent effects, including the endothelial permeability and angiogenic switch, through activation of NADPH oxidases [91,92,93], it is noteworthy that CCM gene loss of function was also shown to lead to NADPH oxidase upregulation [30]. Furthermore and significantly, whereas the PI3K catalytic subunit alpha (PIK3CA) was identified as the crucial isoform that mediates growth factor-dependent ROS production [94], PTEN has been shown to counteract this effect by stimulating the antioxidant role of autophagy [95,96]. Taken together, these considerations suggest that *PTEN* mutations, including the *PTEN* c.959dup p.(Leu320PhefsTer5) frameshift variant in CCM case #4 identified by our study, may affect major redox mechanisms implicated in vascular homeostasis and contribute to CCM disease pathogenesis and severity, thereby opening a new avenue for future research.

## 5. Conclusions

Overall, our NGS analysis allowed to identify causative variants in known CCM genes, as well as variants in other genes that may directly or indirectly contribute to CCM disease pathogenesis and severity, thus providing new genetic insights and suggesting novel avenues for future research aimed at better addressing the emerged complexity of CCM disease pathogenesis. Indeed, while the rapid identification of a germline pathogenic variant is essential to promptly confirm the diagnosis of hereditary CCM disease and guide genetic counselling for at-risk family members, the discovery of risk factors and genetic modifiers that influence CCM disease penetrance and expressivity may significantly contribute to the identification of biomarkers of prognostic and predictive value and the development of novel precision medicine strategies. In particular, despite the shortcoming of a low number of patients analyzed, our identification of *NOTCH3* and *PTEN* variants in CCM cases raises the possibility that the somatic mosaicism of either *NOTCH3* or *PTEN* mutations and their impact on redox mechanisms and oxy-inflammatory responses contribute to CCM disease pathogenesis and severity, thereby supporting the necessity for implemented NGS analysis to enable the sensitive identification of causal genetic variants and effective polygenic risk prediction. In turn, the possibility that both *NOTCH3* and *PTEN* variants contribute to CCM disease pathogenesis and severity is consistent with the recent discovery that NOTCH3 can transactivate PTEN and inhibit the PTEN downstream Akt/mTOR pathway [97], and the parallel identification of CCM causative mutations in genes involved in such a pathway, including *PIK3CA* and *Akt* [14,15,16]. Further analyses in experimental models and large patient cohorts are needed to address the new research perspectives and challenges evoked by our study, including the necessity to combine the identification of potentially significant variants in both causative and modifier genes underlying the broad variability of CCM phenotypes in order to allow effective polygenic risk stratification and personalized treatment for CCM disease.

## Figures and Tables

**Table 1:** Cerebro panel: 23 genes associated with cerebrovascular diseases of genetic origin.

Gene	MIM	Disease
*ABCC6*	603234	Pseudoxanthoma elasticum and arterial calcification generalized of infancy 2 (GACI2)
*ACTA2*	102620	Multisystemic smooth muscle dysfunction syndrome (MSMDS) and aortic aneurysm familial thoracic 6 (AAT6)
*ACVRL1*	601284	Telangiectasia hereditary hemorrhagic type 2 (HHT2) and pulmonary hypertension primary 1 (PPH1)
*APP*	104760	Cerebral amyloid angiopathy app-related (CAA-APP) and Alzheimer’s disease (AD)
*BRCC3*	300617	Moyamoya disease 4 with short stature hypergonadotropic hypogonadism and facial dysmorphism (MYMY4) and T-Cell prolymphocytic leukemia (T-PLL)
*CCM2*	607929	Cerebral cavernous malformations 2 (CCM2) and cerebral cavernous malformations (CCM)
*COL3A1*	120180	Ehlers–Danlos syndrome vascular type (EDSVASC) and polymicrogyria with or without vascular-type Ehlers–Danlos syndrome (PMGEDSV)
*COL4A1*	120130	Retinal arteries tortuosity of (RATOR) and angiopathy hereditary with nephropathy aneurysms and muscle cramps (HANAC)
*COL4A2*	120090	Brain small vessel disease 2 (BSVD2) and intracerebral hemorrhage (ICH)
*ENG*	131195	Telangiectasia hereditary hemorrhagic type 1 (HHT1) and hereditary hemorrhagic telangiectasia (HHT)
*FBN1*	134797	Marfan syndrome (MFS) and stiff skin syndrome (SSKS)
*GLA*	300644	Fabry disease (FD) and rare cardiomyopathy
*HTRA1*	602194	Cerebral arteriopathy autosomal recessive with subcortical infarcts and leukoencephalopathy (CARASIL) and cerebral arteriopathy autosomal dominant with subcortical infarcts and leukoencephalopathy type 2 (CADASIL2)
*JAM3*	606871	Hemorrhagic destruction of the brain subependymal calcification and cataracts (HDBSCC) and Jacobsen syndrome (JBS)
*KRIT1*	604214	Cerebral Cavernous Malformations (CCM) and Cerebral Cavernous Malformations type 1 (CCM1)
*MYH11*	160745	Aortic aneurysm familial thoracic 4 (AAT4) and familial thoracic aortic aneurysm and aortic dissection (FAA)
*MYLK*	600922	Aortic aneurysm familial thoracic 7 (AAT7) and megacystis-microcolon-intestinal hypoperistalsis syndrome (MMIHS)
*NOTCH3*	600276	Cerebral arteriopathy autosomal dominant with subcortical infarcts and leukoencephalopathy type 1 (CADASIL1) and lateral meningocele syndrome (LMNS)
*PDCD10*	609118	Cerebral Cavernous Malformations 3 (CCM3) and Cerebral Cavernous Malformations (CCM)
*SAMHD1*	606754	Aicardi–Goutieres syndrome 5 (AGS5) and Chilblain Lupus 2 (CHBL2)
*TGFBR1*	190181	Multiple Self-Healing Squamous Epithelioma (MSSE) and Loeys–Dietz Syndrome 1 (LDS1)
*TGFBR2*	190182	Loeys–Dietz syndrome 2 (LDS2) and colorectal cancer hereditary nonpolyposis type 6 (HNPCC6)
*TREX1*	606609	Vasculopathy retinal with cerebral leukodystrophy (RVCL) and Aicardi–Goutieres syndrome 1 (AGS1)

**Table 2:** NGS screening of 21 patients affected by Cerebral Cavernous Malformation disease.

ID	Sex	Gene	Ref Seq ^a^	cDNA/ Genomic DNA	Protein	Variant Type	GnomAd v.2.1 Frequency	ACMG Classification	Ref.
1	M	*NOTCH3*	NM_000435.2 (LRG n.a.)	c.2960C>G	p.(Thr987Ser)	Missense	14/215,582	Benign—PM1, PP2, BS1, BP4	[42]
2	M	ND							
3	F	ND							
4 ^b^	M	*PTEN*	NM_000314.8 (LRG_311)	c.959dup	p.(Leu320PhefsTer5)	Frameshift	Not found	Pathogenic—PVS1, PM2, PP3	This report
5	M	ND							
6	M	ND							
7	F	*KRIT1*	NC_000007.13	g.(91715729_91718698)_(91864237_91972337)del		Large genomic deletion	Not found	Pathogenic	[39]
8	F	*PDCD10*	NM_007217.4 (LRG_651)	c.575_576insT	p.(Ser193LysfsTer36)	Frameshift	Not found	Pathogenic—PVS1, PM2, PP3	See ClinVar SCV000808927.1
9	F	ND							
10	M	ND							
11	F	ND							
12	M	*KRIT1*	NM_194456.1 (LRG_650)	c.1664C>T	p.(Ala555Ter)	Splicing	Not found	Pathogenic—PM2, PP1, PP5, PS3, PS4	[43]
13	F	ND							
14	F	ND							
15	F	ND							
16	F	ND							
17	M	ND							
18	M	*CCM2*	NM_031443.4 (LRG_664t1)	c.400delG	p.(Val134CysfsTer22)	Frameshift	Not found	Likely Pathogenic—PVS1, PM2	This report
19	F	*KRIT1*	NM_194456.1 (LRG_650)	c.1057_1060del	p.(Gly353AsnfsTer17)	Frameshift	Not found	Pathogenic—PVS1, PP3, PM2	This report
20	F	*PDCD10*	NM_007217.4 (LRG_651)	c.103C>T	p.(Arg35Ter)	Nonsense	Not found	Pathogenic—PVS1, PP5, PM2, PP3	See ClinVar VCV000372445.5
21	F	ND							

^a^ The MANE reference sequence of the gene is reported. In brackets, we report the Locus Reference Genomic (LRG) record if available (https://www.lrg-sequence.org/, accessed on 8 October 2021). ^b^ Genetic variants in PTEN are currently unrelated to the CCM phenotype; however, they are of potential interest as PTEN plays a critical role in the PI3K/Akt pathway, which has recently been involved in the CCM disease pathogenesis. ND, not detected: no significant SNVs, INDELs and CNVs detected by our NGS analysis. Data reported use the American College of Medical Genetics and Genomics (ACMG) classification to identify the benign or pathological effect of mutations [41]. n.a., not applicable.

## Data Availability

GenBank (https://www.ncbi.nlm.nih.gov/gene, accessed on 8 October 2021); Roche Diagnostics Universal probe Library software (http://www.universalprobelibrary.com, accessed on 8 October 2021) (is no longer available); Locus Reference Genomic (https://www.lrg-sequence.org/, accessed on 8 October 2021); Human Splicing Finder (ver.3; http://www.umd.be/HSF/, accessed on 8 October 2021); MetaDome tool (https://stuart.radboudumc.nl/metadome/, accessed on 8 October 2021).

## Supplementary Materials

The following are available online at https://www.mdpi.com/article/10.3390/antiox11071294/s1. Figure S1: MRI of patient II.3 of family 12. Sequential sagittal (panels A-C) and coronal (panel D) MRI sections showing a cavernoma at the foramen magnum, after the odontoid process, characterized by an elongated shape. Sequential axial MRI sections (panels E-G) showing two additional cavernomas in correspondence of the anterolateral horns of the medulla in D2 and the cauda equina in L3. Sequential transversal (panel H, I) and coronal (panels J-L) MRI section showing lesions in the left superficial white matter of the frontal lobe. Table S1: Quantitative real-time PCR primers and probes for characterization of the KRIT1 genomic deletion in case 7.
