# Supplementary material for: Next-Generation Sequencing Advances the Genetic Diagnosis of Cerebral Cavernous Malformation (CCM)
Source: Antioxidants (Basel). 2022 Jun 29;11(7):1294. doi: 10.3390/antiox11071294 (PMC9311989; doi:10.3390/antiox11071294)
Supplement: Supplementary file 1 [file antioxidants-11-01294-s001.zip › antioxidants-1786205-supplementary.pdf]

Table S1. Quantitative real-time PCR primers and probes for characterization of the *KRIT1* genomic deletion in case 7.

Gene	Ref Seq	Exon / Intron	UPL Probe	Primer F	Primer R	Amplicon (nucleotide)
AKAP9	NM_005751.5	Int 16	#18 (cat. no. 04686918001)	tctttttgtttagaaccgtgagg	aaagaaataaaagcaaacaataaagga	(125 nt) tctttttgtttagaaccgtgaggccatgctttttctgtttatacaattttgagtactaatttactttttaaattcctgctgatattctaaatctaatcctttattgtttgcttttatttcttt
		Int 38	#7 (cat. no. 04685059001)	gaatatagccagtatcccaacagc	agaaaaatcagaacaatgattacctct	(68 nt) gaatatagccagtatcccaacagccccttctcccaagcacatgctcctctgagtaacttagaggtaatcattgttctgatttttct
CYP51A1	NM_000786.3	Ex 1	#52 (cat. no. 04688490001)	accatctgccagcttctctc	ttcactccgtcggaaacact	(110 nt) cccaatgtcatgaatgtcctttaaagattcttacatgttttatatttattattgacatgttttactttgtaggctggtctcgtggtaaaactgttaatgaagctaaatgg
KRIT1	NM_194456.1	Int 19	#54 (cat. no. 04688511001)	cccaatgtcatgaatgtccttta	ccatttagcttcattaacagttttacc	(110 nt) cccaatgtcatgaatgtcctttaaagattcttacatgttttatatttattattgacatgttttactttgtaggctggtctcgtggtaaaactgttaatgaagctaaatgg
		Ex 13	#84 (cat. no. 04689089001)	tctgttgaattgaagcatgga	ttcttgagagagacgcattcc	(72 nt) tctgttgaattgaagcatggaaataataccacagtgcagcagataatggaaggaatgcgtctctctcaagaa
		Int 4	#72 (cat. no. 04688953001)	tgaacagtaaagatgcagtcaaagt	ttgaaaacttttatttccacataatca	(127 nt) tgaacagtaaagatgcagtcaaagtcattcctggcttgccttttaggcactgtatattctaggatagagaacattttatttttttttaattttttcgacatgattatgtggaaataaaagttttcaa
ANKIB1	NM_019004	Ex 6	#22 (cat. no. 04686969001)	ccaacgatcaggtgttcaaa	gcgtgtcccaggcattatac	(62 nt) ccaacgatcaggtgttcaaatgccaactccaccaccaagtgggtataatgcctgggacacgc

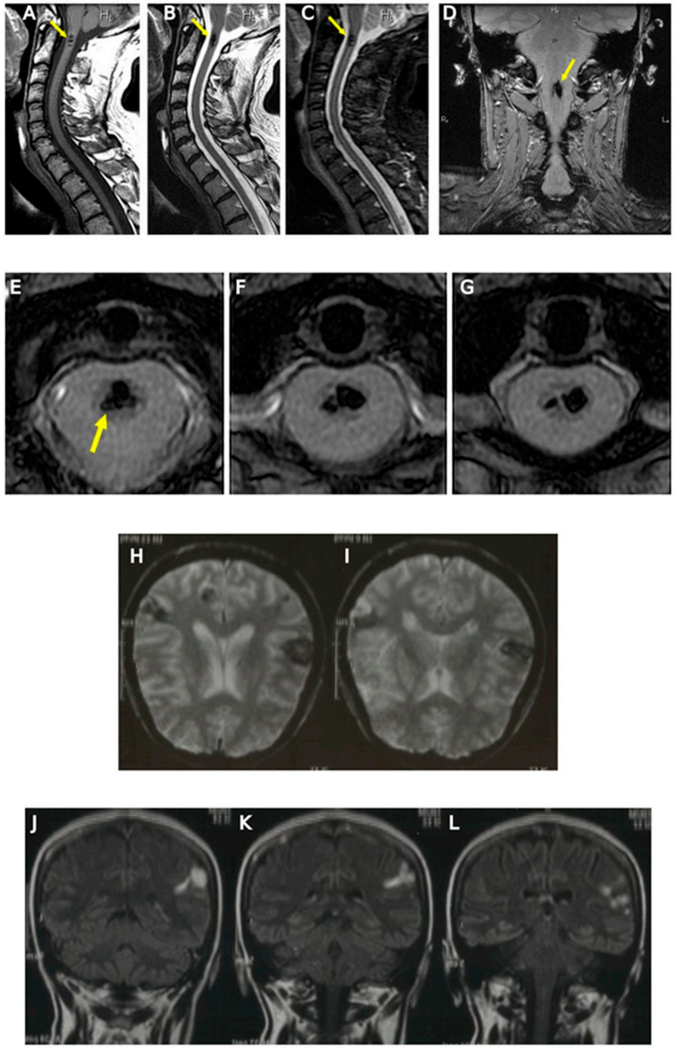

Figure S1. MRI of patient II.3 of family 12. Sequential sagittal (panels A-C) and coronal (panel D) MRI sections showing a cavernoma at the foramen magnum, posterior to the odontoid process, characterized by an elongated shape. Sequential axial MRI sections (panels E-G) showing two additional cavernomas in correspondence of the anterolateral horns of the medulla in D2 and the cauda equina in L3. Sequential transversal (panel H, I) and coronal (panels J-L) MRI section showing lesions in the left superficial white matter of the frontal lobe.
